# Supplementary material for: Sustained rhoptry docking and discharge requires Toxoplasma gondii intraconoidal microtubule-associated proteins
Source: Nat Commun. 2024 Jan 9;15:379. doi: 10.1038/s41467-023-44631-y (PMC10774369; doi:10.1038/s41467-023-44631-y)
Supplement: Supplementary file 1 — Supplementary Information [file 41467_2023_44631_MOESM1_ESM.pdf]

SUPPLEMENTARY INFORMATION

Sustained rhoptry docking and discharge requires *Toxoplasma gondii* intraconoidal microtubule-associated proteins

Nicolas Dos Santos Pacheco[1]*, Albert Tell i Puig[1]*, Amandine Guérin[2]*, Matthew Martinez[3], Bohumil Maco[1], Nicolò Tosetti[1], Estefanía Delgado-Betancourt[1], Matteo Lunghi[1], Boris Striepen[2], Yi-Wei Chang[3,4#] & Dominique Soldati-Favre[1#]

[1] Department of Microbiology and Molecular Medicine, Faculty of Medicine, University of Geneva, Geneva, Switzerland

[2] Department of Pathobiology, School of Veterinary Medicine, University of Pennsylvania, Philadelphia, USA

[3] Department of Biochemistry and Biophysics, Perelman School of Medicine, University of Pennsylvania, Philadelphia, USA

[4] Institute of Structural Biology, Perelman School of Medicine, University of Pennsylvania, Philadelphia, PA, USA

* These authors contributed equally to this work

[#] Correspondence to Dominique.Soldati-Favre@unige.ch and ywc@pennmedicine.upenn.edu

This Supplementary Information includes:

- Supplementary Figures 1-7 and their legends
- Supplementary Discussion

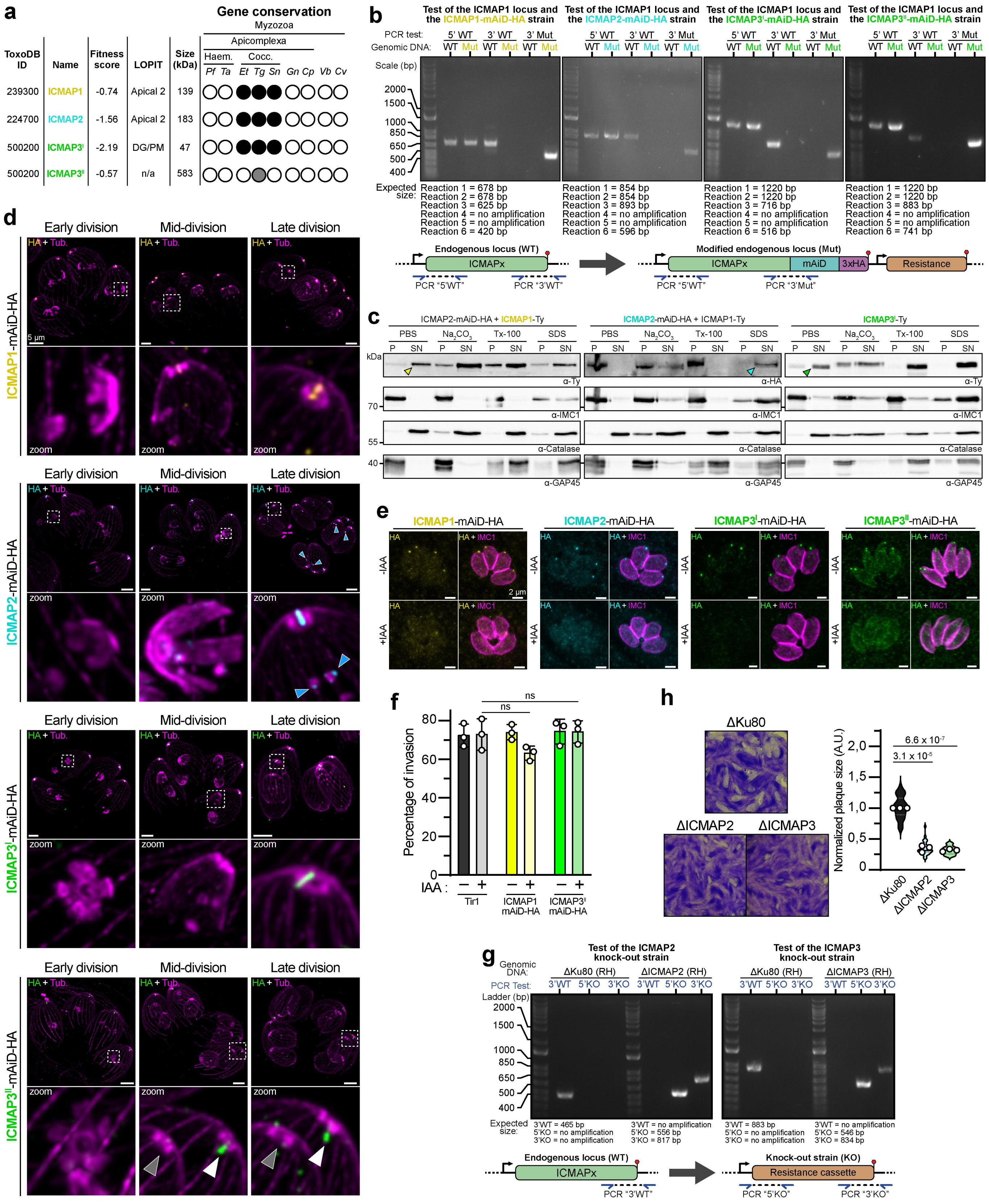

a
Gene conservation
Myzozoa
Apicomplexa
ToxoDB ID
Name
Fitness score
LOPIT
Size (kDa)
Haem.
Cocc.
Pf Ta Et Tg Sn Gn Cp Vb Cv
239300 ICMAP1 -0.74 Apical 2 139
224700 ICMAP2 -1.56 Apical 2 183
500200 ICMAP3I -2.19 DG/PM 47
500200 ICMAP3II -0.57 n/a 583
b
Test of the ICMAP1 locus and the ICMAP1-mAiD-HA strain
Test of the ICMAP1 locus and the ICMAP2-mAiD-HA strain
Test of the ICMAP1 locus and the ICMAP3I-mAiD-HA strain
Test of the ICMAP1 locus and the ICMAP3II-mAiD-HA strain
PCR test:
5' WT
3' WT
3' Mut
Genomic DNA:
WT Mut
Scale (bp)
2000
1500
1000
850
650
500
400
Expected size:
Reaction 1 = 678 bp
Reaction 2 = 678 bp
Reaction 3 = 625 bp
Reaction 4 = no amplification
Reaction 5 = no amplification
Reaction 6 = 420 bp
Reaction 1 = 854 bp
Reaction 2 = 854 bp
Reaction 3 = 893 bp
Reaction 4 = no amplification
Reaction 5 = no amplification
Reaction 6 = 596 bp
Reaction 1 = 1220 bp
Reaction 2 = 1220 bp
Reaction 3 = 716 bp
Reaction 4 = no amplification
Reaction 5 = no amplification
Reaction 6 = 516 bp
Reaction 1 = 1220 bp
Reaction 2 = 1220 bp
Reaction 3 = 883 bp
Reaction 4 = no amplification
Reaction 5 = no amplification
Reaction 6 = 741 bp
Endogenous locus (WT)
ICMAPx
PCR "5'WT"
PCR "3'WT"
Modified endogenous locus (Mut)
ICMAPx
mAiD
3xHA
Resistance
PCR "5'WT"
PCR "3'Mut"
c
ICMAP2-mAiD-HA + ICMAP1-Ty
ICMAP2-mAiD-HA + ICMAP1-Ty
ICMAP3I-Ty
PBS
Na2CO3
Tx-100
SDS
P SN
kDa
70
55
40
α-Ty
α-HA
α-IMC1
α-Catalase
α-GAP45
d
Early division
Mid-division
Late division
HA + Tub.
5 µm
zoom
ICMAP1-mAiD-HA
ICMAP2-mAiD-HA
ICMAP3I-mAiD-HA
ICMAP3II-mAiD-HA
e
ICMAP1-mAiD-HA
ICMAP2-mAiD-HA
ICMAP3I-mAiD-HA
ICMAP3II-mAiD-HA
HA
HA + IMC1
-IAA
+IAA
2 µm
f
Percentage of invasion
0
20
40
60
80
ns
ns
IAA :
– +
Tir1
ICMAP1 mAiD-HA
ICMAP3II mAiD-HA
h
ΔKu80
ΔICMAP2
ΔICMAP3
Normalized plaque size (A.U.)
0
0,5
1,0
1,5
2,0
6.6 x 10-7
3.1 x 10-5
ΔKu80
ΔICMAP2
ΔICMAP3
g
Test of the ICMAP2 knock-out strain
Test of the ICMAP3 knock-out strain
Genomic DNA:
ΔKu80 (RH)
ΔICMAP2 (RH)
ΔKu80 (RH)
ΔICMAP3 (RH)
PCR Test:
3'WT 5'KO 3'KO
Ladder (bp)
2000
1500
1000
850
650
500
400
Expected size:
3'WT = 465 bp
5'KO = no amplification
3'KO = no amplification
3'WT = no amplification
5'KO = 556 bp
3'KO = 817 bp
3'WT = 883 bp
5'KO = no amplification
3'KO = no amplification
3'WT = no amplification
5'KO = 546 bp
3'KO = 834 bp
Endogenous locus (WT)
ICMAPx
PCR "3'WT"
Knock-out strain (KO)
Resistance cassette
PCR "5'KO"
PCR "3'KO"

Supplementary Figure 1 – Additional ICMAP1-2-3 characterization

a- Recapitulative table of ICMAPs properties. Data are extracted from ToxoDB website. Cocc., Coccidia; Haem., Heamosporidia; Pf, *Plasmodium falciparum*; Ta, *Theileria annulata*; Et, *Eimeria tenella*; Tg, *Toxoplasma gondii*; Sn, *Sarcocystis neurona*; Gn, *Gregarina niphandrodes;* Cp, *Cryptosporidium parvum*; Vb, *Vitrella brassicaformis*; Cv, *Chromera velia.* For conservation, ortholog presence (black circle) or absence (open circle) in selected myzozoan taxa is shown (grey circle when the flawed genome annotation does not allow proper conservation assessment). **b-** Integration PCR for the four ICMAPs fused with mAiD-HA cassette. **c-** Solubility of ICMAP1 (left), ICMAP2 (middle) and $ICMAP3^{I}$ (right) in PBS (soluble proteins), Na_2CO_3 (protein complex or cytoskeleton-bound), Tx-100 (membrane proteins), and SDS (control). IMC1, a protein of the alveolin network, is used as a control that should be solubilized in SDS only. Catalase, a soluble protein, is used as a control that should be readily soluble in PBS. GAP45, a membrane-bound protein, is used as a control that should be solubilized by Tx-100 and SDS. Note that for the assessment of $ICMAP3^{I}$ solubility, a specific $ICMAP3^{I}$-Ty strain has been generated. Also, it is important to note that proteins can end up in the soluble and/or insoluble fractions due to aggregation or protein misfolding during handling. P, pellet; SN, Supernatant. **d-** Localization by U-ExM of $ICMAP1\text{-}2\text{-}3^{I}$ at different stages of daughter cell formation. ICMAP2 was also observed associated with centrioles of daughter cells (highlighted by blue arrowheads). For $ICMAP3^{II}$, white arrowheads point to mother cell conoid and grey arrowheads point to daughter cell conoid. Note the petal-like arrangement of the SPMTs in early stages of daughter cells formation. **e-** Downregulation of ICMAP1-, ICMAP2-, $ICMAP3^{I}$- and $ICMAP3^{II}$-mAiD-HA by IFA. **f-** Red-green invasion assay for ICMAP1- and $ICMAP3^{II}$-mAiD-HA strains. Mean ± SD with individual replicates presented (n=3 independent biological replicates) and unpaired two-tailed Student's t-tests were performed where ns if $P > 5 \times 10^{-2}$. **g-** Integration PCR for the two knock-out lines where the endogenous locus was excised and replaced by a resistance cassette (DHFR). **h-** Plaque assay of control, ΔICMAP2 and ΔICMAP3 strains. Quantification of n=3 independent biological triplicate is presented on the right. Mean of the individual replicates are presented as white dots, the median of the total distribution of plaque size is presented as a solid line while the first and third quartiles are presented as dotted lines. Unpaired two-tailed Student's t-tests was performed, and p-value are indicated on the graph.

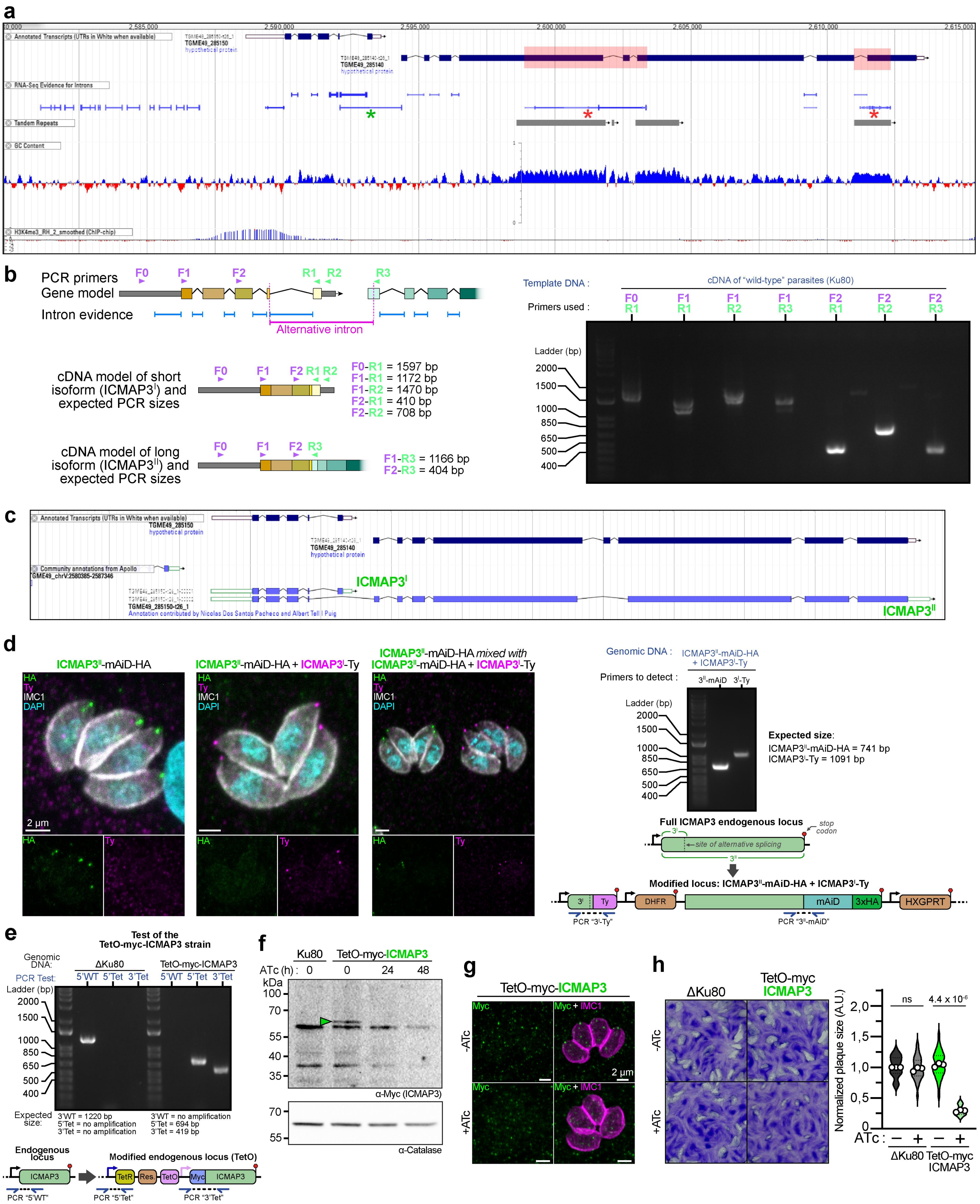

a
Annotated Transcripts (UTRs in White when available)
TGME49_285150
hypothetical protein
TGME49_285140
hypothetical protein
RNA-Seq Evidence for Introns
Tandem Repeats
GC Content
H3K4me3_RH_2_smoothed (ChIP-chip)
b
PCR primers
Gene model
Intron evidence
Alternative intron
cDNA model of short isoform (ICMAP3^I) and expected PCR sizes
F0-R1 = 1597 bp
F1-R1 = 1172 bp
F1-R2 = 1470 bp
F2-R1 = 410 bp
F2-R2 = 708 bp
cDNA model of long isoform (ICMAP3^II) and expected PCR sizes
F1-R3 = 1166 bp
F2-R3 = 404 bp
Template DNA :
cDNA of "wild-type" parasites (Ku80)
Primers used :
Ladder (bp)
c
Annotated Transcripts (UTRs in White when available)
Community annotations from Apollo
Annotation contributed by Nicolas Dos Santos Pacheco and Albert Tell i Puig
ICMAP3^I
ICMAP3^II
d
ICMAP3^II-mAiD-HA
ICMAP3^II-mAiD-HA + ICMAP3^I-Ty
ICMAP3^II-mAiD-HA mixed with ICMAP3^II-mAiD-HA + ICMAP3^I-Ty
HA
Ty
IMC1
DAPI
2 µm
Genomic DNA :
ICMAP3^II-mAiD-HA + ICMAP3^I-Ty
Primers to detect :
Expected size:
ICMAP3^II-mAiD-HA = 741 bp
ICMAP3^I-Ty = 1091 bp
Full ICMAP3 endogenous locus
stop codon
site of alternative splicing
Modified locus: ICMAP3^II-mAiD-HA + ICMAP3^I-Ty
DHFR
mAiD
3xHA
HXGPRT
PCR "3^I-Ty"
PCR "3^II-mAiD"
e
Test of the TetO-myc-ICMAP3 strain
Genomic DNA:
ΔKu80
TetO-myc-ICMAP3
PCR Test:
5'WT 5'Tet 3'Tet
Ladder (bp)
Expected size:
3'WT = 1220 bp
5'Tet = no amplification
3'Tet = no amplification
3'WT = no amplification
5'Tet = 694 bp
3'Tet = 419 bp
Endogenous locus
Modified endogenous locus (TetO)
ICMAP3
TetR
Res
TetO
Myc
PCR "5'WT"
PCR "5'Tet"
PCR "3'Tet"
f
Ku80
TetO-myc-ICMAP3
ATc (h) : 0 0 24 48
kDa
α-Myc (ICMAP3)
α-Catalase
g
TetO-myc-ICMAP3
Myc
Myc + IMC1
-ATc
+ATc
2 µm
h
ΔKu80
TetO-myc ICMAP3
-ATc
+ATc
Normalized plaque size (A.U.)
ns
4.4 x 10^-6
ATc :
ΔKu80
TetO-myc ICMAP3

Supplementary Figure 2 – Splicing variants of ICMAP3

a- Characteristics of TGME49_285150 and TGME49_285140 from ToxoDB (prior version 66). Red highlighted regions and red asterisks corresponds to repeated sequences impairing proper sequencing. Green asterisk corresponds to alternative intron at the origin of the two ICMAP3 isoforms. **b-** Schematization of the alternative intron splicing based on diagnostic PCR (right) performed on wild-type cDNA. Double band are likely due to an unproper melting temperature of the "F1" primer. **c-** Apollo community annotation added to reflect the presence of the two isoforms spanning TGME49_285150 and 285140. **d-** Loss of ICMAP3^{II}-HA staining when ICMAP3^{I} is Ty-tagged at the C-terminus, suggesting a single promoter. As a control for the HA detection, the ICMAP3^{II}-mAID-HA + ICMAP3^{I}-Ty line was mixed with ICMAP3^{II}-mAID-HA on the last picture: a vacuole of ICMAP3^{II}-mAID-HA parasites is seen on the left and a vacuole of ICMAP3^{II}-mAID-HA + ICMAP3^{I}-Ty is seen on the right. Integration PCR on genomic DNA for the ICMAP3^{II}-mAID-HA + ICMAP3^{I}-ty is shown on the right. **e-** Integration PCR for the TetO-myc-ICMAP3 line. The promoter of the TGME49_285150 locus is replaced, affecting both short and long isoforms. **f-** Western blot showing the depletion of ICMAP3 (arrow) upon ATc induction in the TetO-myc-ICMAP3 strain. **g-** The ICMAP3 signal at the ICMTs is loss upon ATc treatment by IFA.**h-** Plaque assay of control and TetO-myc-ICMAP3 strain. Quantification of n=3 independent biological triplicate is presented on the right. Mean of the individual replicates are presented as white dots, the median of the total distribution of plaque size is presented as a solid line while the first and third quartiles are presented as dotted lines. Unpaired two-tailed Student’s t-tests was performed where ns if $P > 5 \times 10^{-2}$.

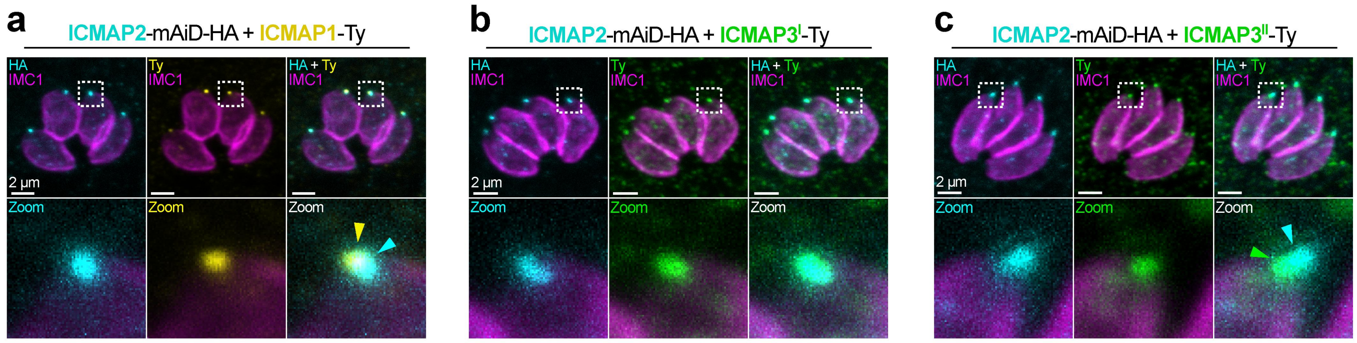

Supplementary Figure 3 – ICMAPs relationship with ICMAP2

a- Partial co-localization of ICMAP1-ICMAP2 by IFA. **b-** Co-localization of ICMAP2-ICMAP3^{I} by IFA. **c-** Partial co-localization of ICMAP2-ICMAP3^{II} by IFA.

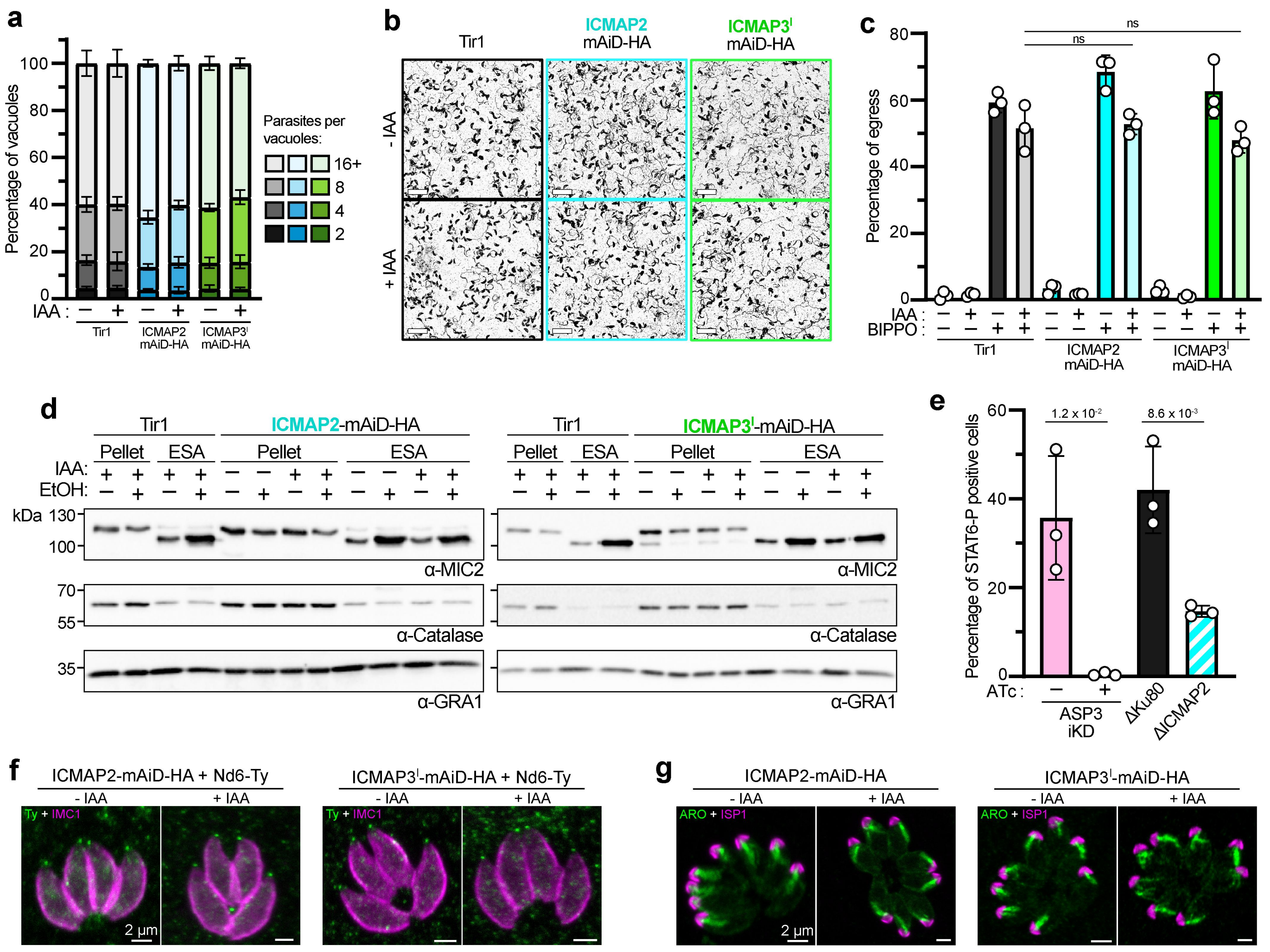

Supplementary Figure 4 – Complementary assays upon ICMAP2 and ICMAP3^{l} depletion

a- Intracellular growth assay after 30h. **b-** ICMAP2- and ICMAP3^{l}-depleted parasites are not impaired in gliding motility as assessed by trails assay. Trails are stained using an anti-SAG1 antibody. Scale bar = 20µm. **c-** ICMAP2- and ICMAP3^{l}-depleted parasites are not impaired in egress stimulated with BIPPO. Mean ± SD with individual replicates (n=3 independent biological replicates) is presented and unpaired two-tailed Student's t-tests were performed where ns if P > 5 x 10-^{2}. **d-** ICMAP2- and ICMAP3^{l}-depleted parasites are not impaired in micronemes secretion. Unprocessed and processed MIC2 can be detected. Catalase is used as lysis control. GRA1 is used as loading control. **e-** Rhoptry secretion is affected upon *icmap2* knock-out (in a ΔKu80 background). Asp3 is used as a control completely blocked in rhoptry secretion in presence of ATc. Mean ± SD with individual replicates (n=3 independent biological replicates) is presented and unpaired two-tailed Student's t-tests were performed and p-values presented on the graph. **f-** Nd6 at the rhoptry secretion apparatus (AV) is not affected upon ICMAP2 or ICMAP3^{l} depletion as seen by IFA. **g-** Assessment of the rhoptry (ARO) organization upon ICMAP2 and ICMAP3^{l} depletion by IFA.

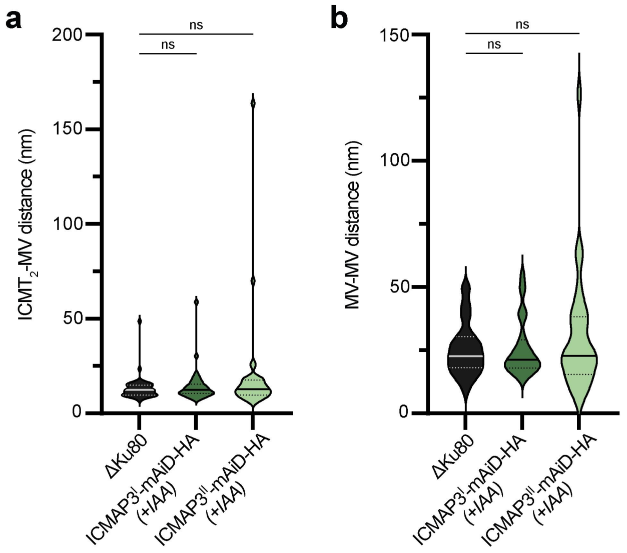

Supplementary Figure 5 – MVs localization in the conoid

a- $ICMT_2$-MV distances were not significantly affected in $ICMAP3^{I}$ and $ICMAP3^{II}$-depleted parasites. For the three conditions presented n = 45, 37 and 39 respectively. Unpaired two-tailed Student's t-tests was performed where ns if $P > 5 \times 10^{-2}$. **b-** MV-MV distances were not significantly affected in $ICMAP3^{I}$ and $ICMAP3^{II}$-depleted parasites. For the three conditions presented n = 35, 27 and 29 respectively. Unpaired two-tailed Student's t-tests was performed where ns if $P > 5 \times 10^{-2}$.

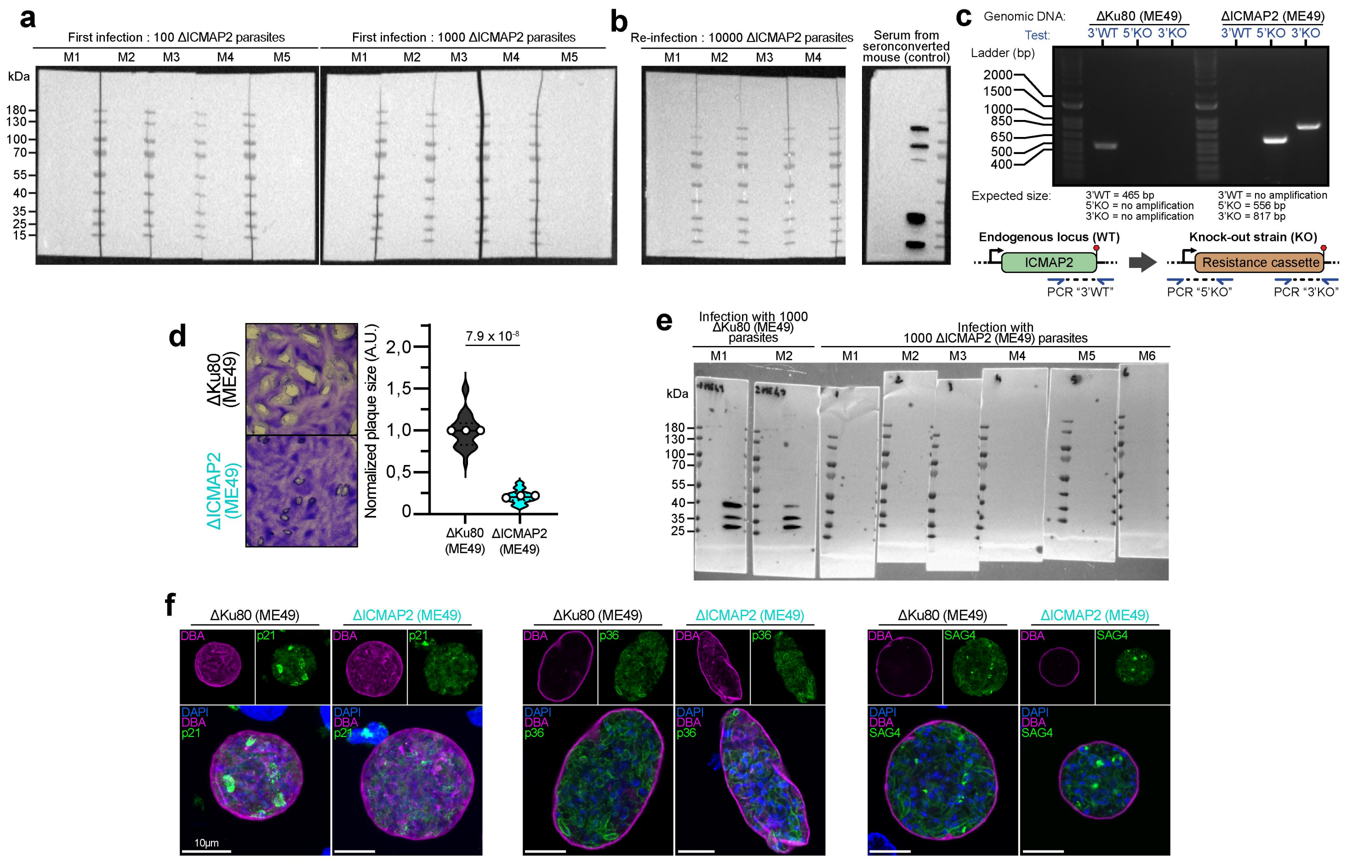

Supplementary Figure 6 – Seroconversion and cyst formation in ICMAP2-depleted parasites

a- Serum test of mice infected with 100 (n=5) or 10000 (n=5) parasites. Blood test was performed at day 16 post-infection. **b-** Serum test of mice infected with 10000 (n=4) parasites for the re-infection experiment. Blood test was performed at day 30 post-reinfection. **c-** Integration PCR for the ICMAP2 knock-out lines (ME49 background) where the endogenous locus was excised and replaced by a resistance cassette (DHFR). **d-** Plaque assay of control and ΔICMAP2 (ME49 background) strains. Quantification of n=3 independent biological triplicate is presented on the right. Mean of the individual replicates are presented as white dots, the median of the total distribution of plaque size is presented as a solid line while the first and third quartiles are presented as dotted lines. Unpaired two-tailed Student's t-tests was performed, and p-value are indicated on the graph. **e-** Serum test of mice infected with 1000 ME49 ΔKu80 (n=2) or 1000 ME49 ΔICMAP2 (n=6) parasites. Blood test was performed at day 16 post-infection. **f-** IFA of cysts with different bradyzoite markers such as p21, p36 and SAG4.

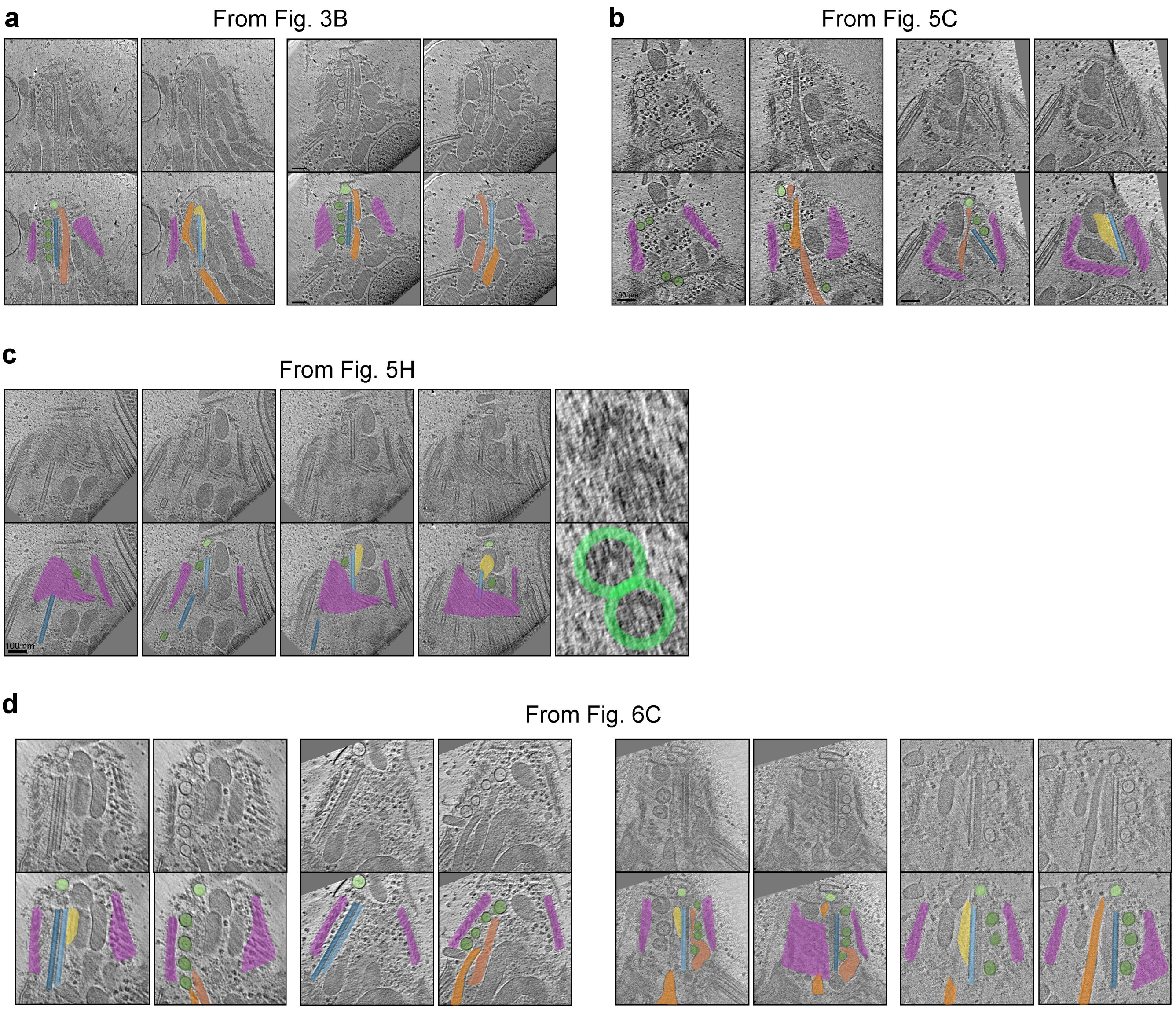

Supplementary Figure 7 – Raw tomograms presented in this study

a- Tomograms slices of Fig. 3B without overlay. **b-** Tomograms slices of Fig. 5C without overlay. **c-** Tomogram slices of Fig. 5H without overlay. **d-** Tomogram slices of Fig. 6C without overlay.

Supplementary Discussion

While the ToxoDB version 66 (published in November 2023) named the ICMAP3 locus as TGME49_500200, prior versions described instead the presence of two separate ORFs, namely TGME49_285140 and TGME49_285150 (Supplementary Fig. 2A).

Analysis of the TGME49_285140 locus using ToxoDB (prior version 66) led us to question the veracity of the displayed gene model (Supplementary Fig. 2A). Chip-chip and Chip-seq datasets did not show the histone modifications indicative of promoter regions at the predicted start of the coding sequence (Supplementary Fig. 2A). In addition, the RNA-seq evidence did not support introns in at least two regions (chrV:2,599,000 to 2,306,375 and chrV:2,611,250 to 2,612,375)(Supplementary Fig. 2A – red regions and asterisks). This was likely due to tandem repeats and high GC content impeding proper sequencing and assembly of the gene. In contrast, the upstream gene, TGME49_285150, appeared well annotated and apparently possessed an alternative intron spanning the two models (Supplementary Fig. 2A – green asterisk), resulting in a single open reading frame (ORF). Those data therefore suggest that TGME49_285150 and TGME49_285140 were in fact part of a single gene. To accurately annotate this locus, primers were designed to amplify and sequence the mRNA transcripts covering both loci (Supplementary Fig. 2B). The results demonstrated that this ORF codes for a short and a long isoform resulting from alternative splicing. Accordingly, both isoforms were annotated in the ToxoDB through the Apollo community platform [1, 2] (Supplementary Fig. 2C). The ToxoDB version 66 now name this locus TGME49_500200.

To confirm the existence of two isoforms, a Ty-tag was introduced at the C-terminus of ICMAP3^{I} in the ICMAP3^{II}-mAiD-HA strain. In this case, ICMAP3^{I} tagging led to the ICMAP3^{II} signal not being detectable anymore. This likely indicates that the introduction of the Ty-DHFR cassette at C-terminus of the short isoform disrupted the alternative intron, demonstrating that both isoforms share the same promoter (Supplementary Fig. 2D).

To simultaneously delete both ICMAP3 isoforms, we engineered a version of the gene where the native promoter was replaced by a tetracycline-repressible promoter (TetO)[3] and tagged at the N-terminus with a Myc epitope (TetO-myc-ICMAP3) (Supplementary Fig. 2E). Western blot analysis confirmed the presence of the ICMAP3^{I} isoform with an apparent molecular weight of 60-65kDa while ICMAP3^{II} remained undetectable (Supplementary Fig. 2F). This Myc signal was lost following 24h of ATc treatment (Supplementary Fig. 2F-G) and a reduction of ~75% in plaque size was observed by plaque assay (Supplementary Fig. 2H).

1. Dunn, N. A., Unni, D. R., Diesh, C., Munoz-Torres, M., Harris, N. L., Yao, E., Rasche, H., Holmes, I. H., Elsik, C. G., and Lewis, S. E., *Apollo: Democratizing genome annotation.* PLoS Comput Biol, **2019**. 15(2): p. e1006790.
2. Harb, O. S. and Roos, D. S., *ToxoDB: Functional Genomics Resource for Toxoplasma and Related Organisms.* Methods Mol Biol, **2020**. 2071: p. 27-47.
3. Meissner, M., Brecht, S, Bujard, H, and Soldati-Favre, D.,*Modulation of myosin A expression by a newly established tetracycline repressor-based inducible system in Toxoplasma gondii.* Nucleic Acid Research, **2001**. 29(22).
