# Supplementary material for: Sustained rhoptry docking and discharge requires Toxoplasma gondii intraconoidal microtubule-associated proteins
Source: Nat Commun. 2024 Jan 9;15:379. doi: 10.1038/s41467-023-44631-y (PMC10774369; doi:10.1038/s41467-023-44631-y)
Supplement: Supplementary file 3 — Description of Additional Supplementary Files [file 41467_2023_44631_MOESM3_ESM.docx]

**Description of additional supplementary files**

**Title:** Supplementary Data 1

**Description:** Detail of all primers, plasmids, parasites strains, antibodies, and supplies used in this study.

**Title:** Supplementary Movie 1

**Description:** Tomogram and 3D reconstruction of a WT apical complex.

**Title:** Supplementary Movie 2

**Description:** Tomogram and 3D reconstruction of an ICMAP1-depleted parasite apical complex.

**Title:** Supplementary Movie 3

**Description:** Serial sections (FIB-SEM) and 3D reconstruction of a WT parasite.

**Title:** Supplementary Movie 4

**Description:** Serial sections (FIB-SEM) and 3D reconstruction of an ΔICMAP2 parasite.

**Title:** Supplementary Movie 5

**Description:** Tomogram and 3D reconstruction of three ICMAP2-depleted parasite apical complex.

**Title:** Supplementary Movie 6

**Description:** Tomogram and 3D reconstruction of two ICMAP3^I^-depleted parasite apical complex.

**Title:** Supplementary Movie 7

**Description:** Tomogram and 3D reconstruction of two ICMAP3^II^-depleted parasite apical complex.
