# Supplementary material for: Sustained rhoptry docking and discharge requires Toxoplasma gondii intraconoidal microtubule-associated proteins
Source: Nat Commun. 2024 Jan 9;15:379. doi: 10.1038/s41467-023-44631-y (PMC10774369; doi:10.1038/s41467-023-44631-y)
Supplement: Supplementary file 12 — Reporting Summary [file 41467_2023_44631_MOESM12_ESM.pdf]

nature portfolio

Corresponding author(s): Dominique Soldati-Favre

Last updated by author(s): Dec 18, 2023

Reporting Summary

Nature Portfolio wishes to improve the reproducibility of the work that we publish. This form provides structure for consistency and transparency in reporting. For further information on Nature Portfolio policies, see our Editorial Policies and the Editorial Policy Checklist.

Statistics

For all statistical analyses, confirm that the following items are present in the figure legend, table legend, main text, or Methods section.

n/a	Confirmed	
☐	☒	The exact sample size (*n*) for each experimental group/condition, given as a discrete number and unit of measurement
☐	☒	A statement on whether measurements were taken from distinct samples or whether the same sample was measured repeatedly
☐	☒	The statistical test(s) used AND whether they are one- or two-sided<br>*Only common tests should be described solely by name; describe more complex techniques in the Methods section.*
☒	☐	A description of all covariates tested
☒	☐	A description of any assumptions or corrections, such as tests of normality and adjustment for multiple comparisons
☐	☒	A full description of the statistical parameters including central tendency (e.g. means) or other basic estimates (e.g. regression coefficient) AND variation (e.g. standard deviation) or associated estimates of uncertainty (e.g. confidence intervals)
☐	☒	For null hypothesis testing, the test statistic (e.g. *F*, *t*, *r*) with confidence intervals, effect sizes, degrees of freedom and *P* value noted<br>*Give P values as exact values whenever suitable.*
☒	☐	For Bayesian analysis, information on the choice of priors and Markov chain Monte Carlo settings
☒	☐	For hierarchical and complex designs, identification of the appropriate level for tests and full reporting of outcomes
☒	☐	Estimates of effect sizes (e.g. Cohen's *d*, Pearson's *r*), indicating how they were calculated

Our web collection on statistics for biologists contains articles on many of the points above.

Software and code

Policy information about availability of computer code

Data collection	Light microscopy images were acquired using the LasX Software (Leica, version 3.7.0).<br>Electron microscopy images were acquired with the iTEM software (Olympus Imaging system; version 5.2).<br>Western Blot gels and membrane were captured using ImageLab (BioRad, version 1.0).<br>Cryo-ET tilt series data were acquired using SerialEM (version 3.8)
Data analysis	Statistical analysis and graph generation were performed with Prism 9 (GraphPad; version 9).<br>Light microscopy and electron microscopy images were modified using ImageJ (NIH, version 1.53c).<br>Tomogram reconstruction, segmentation, and modeling were performed using IMOD (version 4.11.5).<br>Segmentation and modeling were performed using UCSF ChimeraX (version 1.1).<br>Subtomogram averaging were performed using R2019a and Dynamo (version 1.1.509).

For manuscripts utilizing custom algorithms or software that are central to the research but not yet described in published literature, software must be made available to editors and reviewers. We strongly encourage code deposition in a community repository (e.g. GitHub). See the Nature Portfolio guidelines for submitting code & software for further information.

Data

Policy information about availability of data

All manuscripts must include a data availability statement. This statement should provide the following information, where applicable:

- Accession codes, unique identifiers, or web links for publicly available datasets
- A description of any restrictions on data availability
- For clinical datasets or third party data, please ensure that the statement adheres to our policy

All data are available within the paper and its supplementary information. Sequences used in this study have been obtained from VEuPathDB (https://veupathdb.org/) and ToxoDB (https://toxodb.org). All biological materials and data are available from the author upon request. Source data are provided with this paper. Representative tomograms showing an apical end of ICMAP1-, ICMAP2-, ICMAP3I-, and ICMAP3II-depleted T. gondii are available in the Electron Microscopy Data Bank (EMDB) under accession codes EMD-42118 [https://www.ebi.ac.uk/pdbe/entry/emdb/EMD-42118], EMD-42119 [https://www.ebi.ac.uk/pdbe/entry/emdb/EMD-42119], EMD-42120 [https://www.ebi.ac.uk/pdbe/entry/emdb/EMD-42120], and EMD-42121 [https://www.ebi.ac.uk/pdbe/entry/emdb/EMD-42121], respectively.

Research involving human participants, their data, or biological material

Policy information about studies with human participants or human data. See also policy information about sex, gender (identity/presentation), and sexual orientation and race, ethnicity and racism.

Reporting on sex and gender	Not applicable
Reporting on race, ethnicity, or other socially relevant groupings	Not applicable
Population characteristics	Not applicable
Recruitment	Not applicable
Ethics oversight	Not applicable

Note that full information on the approval of the study protocol must also be provided in the manuscript.

Field-specific reporting

Please select the one below that is the best fit for your research. If you are not sure, read the appropriate sections before making your selection.

☒ Life sciences ☐ Behavioural & social sciences ☐ Ecological, evolutionary & environmental sciences

For a reference copy of the document with all sections, see nature.com/documents/nr-reporting-summary-flat.pdf

Life sciences study design

All studies must disclose on these points even when the disclosure is negative.

Sample size	Sample size were chosen based on previously published litterature in the field. All experiments were performed with enough biological replicates (at least 3) to allow relevant statistical analysis. For cryo-ET, sample size is described for each sample/conditions in the text.
Data exclusions	No data were excluded from the analysis presented in this study.
Replication	All experiments were performed with enough independant biological replicate (at least 3). All results were successfully replicated. Biological replicates are represented on each graph when possible.
Randomization	The study does not involve human clinical trials or other experiments that require randomization. To avoid bias, some experiments were subject to blinding (see below).
Blinding	Some experiments that can be bias by knowledge of the samples (injection/invasion assay) were performed in a blind manner. IFA slides were mounted by a third party and labelled randomly. The person counting the assay did not know which condition was counted before the compiling of the results and the revealing of the conditions by the third party. Otherwise the experiments were not performed in a blinded manner as they are less subject to bias.

Reporting for specific materials, systems and methods

We require information from authors about some types of materials, experimental systems and methods used in many studies. Here, indicate whether each material, system or method listed is relevant to your study. If you are not sure if a list item applies to your research, read the appropriate section before selecting a response.

Materials & experimental systems

n/a	Involved in the study	
	x	Antibodies
	x	Eukaryotic cell lines
x		Palaeontology and archaeology
	x	Animals and other organisms
x		Clinical data
x		Dual use research of concern
x		Plants

Methods

n/a	Involved in the study	
x		ChIP-seq
x		Flow cytometry
x		MRI-based neuroimaging

Antibodies

Antibodies used

All antibodies used in this study are listed in the Supplementary Data 1 (in the "Antibodies" section).

For primary antibodies: Rat anti-HA (Roche, #ROAHAHA clone 3F10) ; Mouse anti-HA (BioLegend, #16B12) ; Rabbit anti-Ty (gift from Chris Tonkin, WEHI) ; Mouse anti-Ty (clone BB2, hybridoma produced in house) ; Mouse anti-Myc (clone 9E10, hybridoma produced in house) ; Rabbit anti-GAP45 (Plattner et al., 2008) ; Guinea Pig anti-alpha tubulin (Geneva Antibody Facility, #AA345) ; Guinea Pig anti-beta tubulin (Geneva Antibody Facility, #AA344) ; Mouse anti-acetylated tubulin (Santa Cruz, #sc23950) ; Rabbit anti-IMC1 (Frénal et al., 2014) ; Mouse anti-MIC2 (gift from J-F Dubremetz, University of Montpellier, hybridoma produced in house) ; Rabbit anti-GRA1 (MyBioSource, #MBS1493292) ; Mouse anti-GRA3 (gift from J-F Dubremetz, University of Montpellier, hybridoma T6.2H11 produced in house) ; Mouse anti-SAG1 (Sibley et al., 1995) ; Rabbit anti-SAG1 (Kim et al., 1993) ; Rabbit anti-catalase (Ding et al., 2000) ; Rabbit anti-ARO (Mueller et al. 2013) ; Rabbit anti-phosphoSTAT6 (Cell Signalling, #9361) ; Mouse anti-actin (Herm-Götz et al., 2002) ; Mouse anti-ISP1 (Beck et al., 2010) ; Mouse anti-p36 (Soete et al. 1993) ; Mouse anti-p21 (Soete et al. 1993) ; Mouse anti-SAG4 (Odberg-Ferragut et al. 1996) ; Mouse anti-BAG1 (Kannan et al. 2021).

For secondary antibodies: Goat anti-guinea pig IgG - AlexaFluor 405 (Abcam, #ab175678) ; Goat anti-mouse IgG - AlexaFluor 488 (Invitrogen, #A11029) ; Goat anti-rabbit IgG - AlexaFluor 488 (Invitrogen, #A11008) ; Goat anti-rat IgG - AlexaFluor 488 (Invitrogen, #A21208) ; Goat anti-guinea pig IgG - AlexaFluor 594 (Invitrogen, #A11076) ; Goat anti-mouse IgG - AlexaFluor 594 (Invitrogen, #A11032) ; Goat anti-rabbit IgG - AlexaFluor 594 (Invitrogen, #A11012) ; Goat anti-rat IgG - AlexaFluor 594 (Invitrogen, #A21209) ; Goat anti-rabbit IgG - AlexaFluor 647 (Invitrogen, #A21244) ; Goat anti-rabbit IgG - HRP (Sigma-Aldrich, #A8275) ; Goat anti-mouse IgG - HRP (Sigma-Aldrich, #A5278) ; DBA-rhodamines (lectin) (Vector laboratories, #RL-1032-2).

Validation

No unpublished antibodies were used in this study. No antibodies were generated for this study. All antibodies were validated in previous publications or by the manufacturer :
- Rat anti-HA (Roche, #ROAHAHA clone 3F10) : validated for IFA and WB by the manufacturer.
- Mouse anti-HA (BioLegend, #16B12) : validated for IFA and WB by the manufacturer.
- Rabbit anti-Ty (gift from Chris Tonkin, WEHI) : Uboldi, Alessandro D et al. “Regulation of Starch Stores by a Ca(2+)-Dependent Protein Kinase Is Essential for Viable Cyst Development in Toxoplasma gondii.” Cell host & microbe vol. 18,6 (2015): 670-81. doi:10.1016/j.chom.2015.11.004
- Mouse anti-Ty (clone BB2) : Bastin, P et al. “A novel epitope tag system to study protein targeting and organelle biogenesis in Trypanosoma brucei.” Molecular and biochemical parasitology vol. 77,2 (1996): 235-9. doi:10.1016/0166-6851(96)02598-4
- Mouse anti-Myc (clone 9E10, hybridoma produced in house) : Evan, Gerard I et al. “Isolation of monoclonal antibodies specific for human c-myc proto-oncogene product.” Molecular and cellular biology vol. 5,12 (1985): 3610-6. doi:10.1128/mcb.5.12.3610-3616.1985
- Rabbit anti-GAP45 : Plattner et al. "Toxoplasma Profilin Is Essential for Host Cell Invasion and TLR11-Dependent Induction of an Interleukin-12 Response" Cell Host Microbe, vol. 3, issue 2, 77-87. doi: 10.1016/j.chom.2008.01.001
- Guinea Pig anti-alpha tubulin (Geneva Antibody Facility, #AA345) : Tosetti, Nicolò et al. “Essential function of the alveolin network in the subpellicular microtubules and conoid assembly in Toxoplasma gondii.” eLife vol. 9 e56635. 7 May. 2020, doi:10.7554/eLife.56635
- Guinea Pig anti-beta tubulin (Geneva Antibody Facility, #AA344) : Tosetti, Nicolò et al. “Essential function of the alveolin network in the subpellicular microtubules and conoid assembly in Toxoplasma gondii.” eLife vol. 9 e56635. 7 May. 2020, doi:10.7554/eLife.56635
- Mouse anti-acetylated tubulin (Santa Cruz, #sc23950) : validated for IFA and WB by the manufacturer.
- Rabbit anti-IMC1 : Mann, T, and C Beckers. “Characterization of the subpellicular network, a filamentous membrane skeletal component in the parasite Toxoplasma gondii.” Molecular and biochemical parasitology vol. 115,2 (2001): 257-68. doi:10.1016/s0166-6851(01)00289-4
- Mouse anti-MIC2 (gift from J-F Dubremetz, University of Montpellier) : Achbarou, A et al. “Characterization of microneme proteins of Toxoplasma gondii.” Molecular and biochemical parasitology vol. 47,2 (1991): 223-33. doi:10.1016/0166-6851(91)90182-6
- Rabbit anti-GRA1 (MyBioSource, #MBS1493292) : validated for IFA and WB by the manufacturer.
- Mouse anti-GRA3 (T6.2H11) : Achbarou, A et al. “Differential targeting of dense granule proteins in the parasitophorous vacuole of Toxoplasma gondii.” Parasitology vol. 103 Pt 3 (1991): 321-9. doi:10.1017/s0031182000059837
- Mouse anti-SAG1 : Couvreur, G et al. “Surface antigens of Toxoplasma gondii.” Parasitology vol. 97 (Pt 1) (1988): 1-10. doi:10.1017/s0031182000066695
-Rabbit anti-SAG1 : Kim, K et al. “Gene replacement in Toxoplasma gondii with chloramphenicol acetyltransferase as selectable marker.” Science (New York, N.Y.) vol. 262,5135 (1993): 911-4. doi:10.1126/science.8235614
- Rabbit anti-catalase : Ding, M et al. “Toxoplasma gondii catalase: are there peroxisomes in toxoplasma?.” Journal of cell science vol.

113 (Pt 13) (2000): 2409-19.
- Rabbit anti-ARO : Mueller, Christina et al. “The Toxoplasma protein ARO mediates the apical positioning of rhoptry organelles, a prerequisite for host cell invasion.” Cell host & microbe vol. 13,3 (2013): 289-301. doi:10.1016/j.chom.2013.02.001
- Rabbit anti-phosphoSTAT6 (Cell Signalling, #9361) : : validated for IFA and WB by the manufacturer.
- Mouse anti-actin : Herm-Götz, Angelika et al. “Toxoplasma gondii myosin A and its light chain: a fast, single-headed, plus-enddirected motor.” The EMBO journal vol. 21,9 (2002): 2149-58. doi:10.1093/emboj/21.9.2149
- Mouse anti-ISP1 : Beck, Josh R et al. “A novel family of Toxoplasma IMC proteins displays a hierarchical organization and functions in coordinating parasite division.” PLoS pathogens vol. 6,9 e1001094. 9 Sep. 2010, doi:10.1371/journal.ppat.1001094).
- Mouse anti-p21 : Soete, Martine et al. "Toxoplasma gondii: kinetics of bradyzoite-tachyzoite interconversion in vitro." Exp Parasitol. 1993 May;76(3):259-64. doi: 10.1006/expr.1993.1031. PMID: 7684705.
- Mouse anti-p36 : Soete, Martine et al. "Toxoplasma gondii: kinetics of bradyzoite-tachyzoite interconversion in vitro." Exp Parasitol. 1993 May;76(3):259-64. doi: 10.1006/expr.1993.1031. PMID: 7684705.
- Mouse anti-SAG4 : Odberg-Ferragut, Carmen et al. “Molecular cloning of the Toxoplasma gondii sag4 gene encoding an 18 kDa bradyzoite specific surface protein.” Molecular and biochemical parasitology vol. 82,2 (1996): 237-44. doi:10.1016/0166-6851(96)02740-5
- Mouse anti-BAG1 : Kannan G, et al. "Acquisition of Host Cytosolic Protein by Toxoplasma gondii Bradyzoites." mSphere. 2021;6(1):e00934-20. Published 2021 Jan 27. doi:10.1128/mSphere.00934-20

Eukaryotic cell lines

Policy information about cell lines and Sex and Gender in Research

Cell line source(s)	Human Foreskin Fibroblasts (HFF, ATCC, CRL1634)<br>T. gondii Tir1 expressing line (generation of new mAID strains) was provided by Pr. Sibley (Washington university) : Brown, Kevin M et al. “Plasma Membrane Association by N-Acylation Governs PKG Function in Toxoplasma gondii.” mBio vol. 8,3 e00375-17. 2 May. 2017, doi:10.1128/mBio.00375-17
Authentication	Cell lines were authenticated by the individual providers.
Mycoplasma contamination	All T. gondii parental lines and host cells were tested negative for Mycoplasma infection by IFA. Following transfections, T. gondii transgenic lines were not tested for mycoplasma infection.
Commonly misidentified lines (See ICLAC register)	None to be reported.

Animals and other research organisms

Policy information about studies involving animals; ARRIVE guidelines recommended for reporting animal research, and Sex and Gender in Research

Laboratory animals	The study involved a total of fifteen 7-weeks old female CD1 mice (Charles River).
Wild animals	No wild animals were used in the study.
Reporting on sex	Only female mice were used.
Field-collected samples	No field-collected samples were used in the study.
Ethics oversight	All animal experiments were conducted with the authorization numbers GE3-20 and GE272, according to the guidelines and regulations issued by the Swiss Federal Veterinary Office. The authorizations were issued through the director of the animal experimentation department of the University of Geneva (Daniele Roppolo) and approved by the cantonal veterinary officer (Dr. Michel Rérat).

Note that full information on the approval of the study protocol must also be provided in the manuscript.
